# *BCR-ABL1*-positive acute lymphoblastic leukemia following successful treatment of acute promyelocytic leukemia: case report

**DOI:** 10.3389/fphar.2023.1141311

**Published:** 2023-06-16

**Authors:** Shuang Fu, Mengqi Li, Hongtao Wang

**Affiliations:** ^1^ Department of Hematology Laboratory, Shengjing Hospital of China Medical University, Shenyang, China; ^2^ Department of Hematology, Shengjing Hospital of China Medical University, Shenyang, China

**Keywords:** acute promyelocytic leukemia, acute lymphoblastic leukemia, PML-RARα, BCR-ABL1, secondary malignancy

## Abstract

Acute promyelocytic leukemia (APL) is currently considered a disease with a higher cure rate. And cases of secondary malignant tumors following successful APL treatment are rare. Here we described a rare case of a 29-year-old man who was treated for APL in 2019 and developed *BCR-ABL1*-positive acute lymphoblastic leukemia 2 years later. The patient responded well to tyrosine kinase inhibitors and chemotherapy, and achieved a molecular remission. Although APL usually has a good prognosis, the prognosis of its secondary malignancies is uncertain. There are no effective measures to prevent the occurrence of secondary tumors. Continuing to increase the monitoring frequency of laboratory tests, especially the molecular biomarkers, is essential for the diagnosis and treatment of secondary malignancies after the patients achieving complete remission.

## Introduction

Acute promyelocytic leukemia (APL) is a distinct type of acute myeloid leukemia (AML), which is designated as M3 subtype by French-American-British classification (Krause, 2000). It is characterized by a large number of abnormal promyelocytic cells in the bone marrow, accompanied with the typical chromosomal translocation t(15; 17) (q22; q12-21). As a result, a fusion gene of the promyelocytic leukemia (*PML*) gene and the retinoic acid receptor alpha (*RARα*) gene was formed by this chromosomal abnormality (Tan et al., 2021; Yilmaz et al., 2021). The application of target specific agents, all-trans retinoic acid (ATRA) and arsenic trioxide (ATO) with or without chemotherapy, is an internationally recognized standard therapy regime for APL patients (Ferrara et al., 2022; Jamy et al., 2022). Despite the success of ATRA and ATO in the treatment of patients with APL, secondary malignant tumors after complete remission (CR) of patients are worthy of attention. *BCR-ABL1* fusion protein has two major isoforms, *BCR-ABL1* (p190) and *BCR-ABL1* (p210). And the *BCR-ABL1* (p190) mainly occurs in acute lymphoblastic leukemia (ALL) patients (El Fakih et al., 2018; Adnan-Awad et al., 2021). Here, we reported a rare patient who developed secondary *BCR-ABL1* (p190)-positive ALL following successful treatment of APL, and provide a literature review to summarize the characteristics of this subset of patients.

## Case report

**FIGURE 1:**
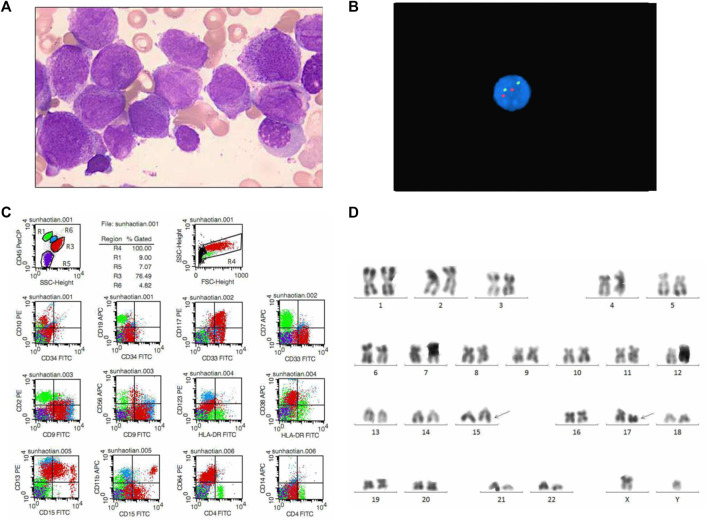
Laboratory results of the patient at diagnosis. **(A)** Morphologic evaluation of bone marrow (Wright–Giemsa stain, × 1000). **(B)** FISH results of BCR-ABL1 in bone marrow. **(C)** Flow cytometry results of bone marrow. R1, lymphocytes; R3, promyeloblasts; R4, total cells; R5, erythrocytoblasts; R6, monocytes. **(D)** Karyotype analysis results of bone marrow.

**FIGURE 2:**
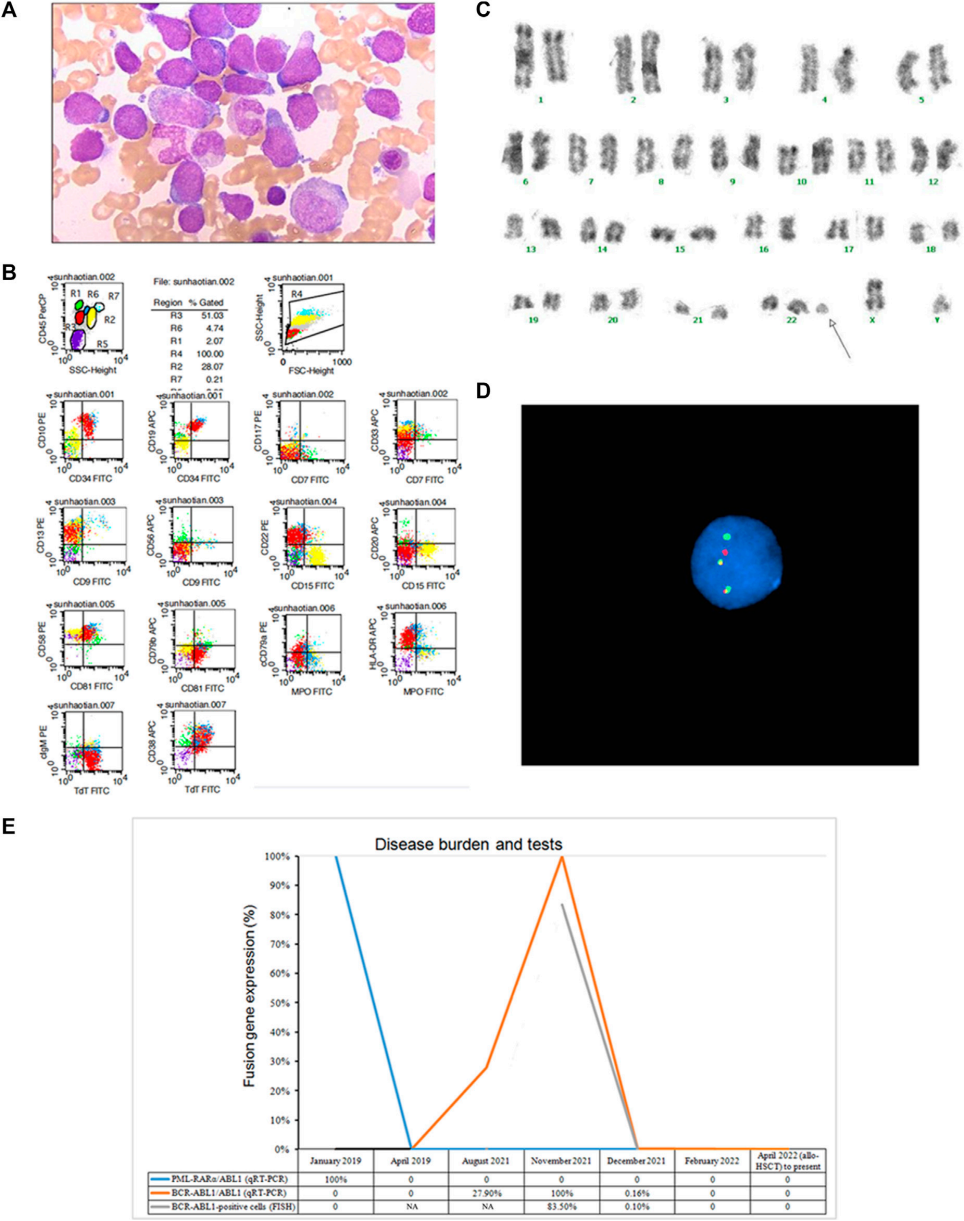
Laboratory results of the patient at secondary ALL and gene expression curve. **(A)** Morphologic evaluation of bone marrow (Wright–Giemsa stain, × 1000). **(B)** Flow cytometry results of bone marrow. R1, lymphocytes; R2, neutrophils; R3, prolymphocytes; R4, total cells; R5, erythrocytoblasts; R6, monocytes; R7, eosinophils. **(C)** Karyotype analysis results of bone marrow. **(D)** FISH results of bone marrow. **(E)** Gene expression curve of the patient by RT-PCR and FISH.

A 29-year-old man was admitted to our hospital for pancytopenia and clustered petechiae and ecchymoses on his left arm in January 2019. The laboratory features of the patient were shown in Table 1. Complete blood count at diagnosis showed white blood cell (WBC) counts 1.0 × 10^9^/L, hemoglobin level 95.0 g/L, platelet counts 20.0 × 10^9^/L. The bone marrow (BM) morphologic evaluation revealed 80.4% typical promyelocytes (Figure 1A). Bone marrow pathology analysis showed a large number of blasts in the hematopoietic tissue (Figure 1B). Immunophenotyping studies of BM cells revealed that the blasts expressed CD45dim, CD117, CD33, CD38, CD13, CD64 and CD15; no lymphoid antigens were expressed (Figure 1C). Cytogenetic analysis result showed an abnormal karyotype of t(15; 17) (q22; q21) (Figure 1D), and *PML-RARa* fusion gene was also detected in the patient’s bone marrow cells. *BCR-ABL1* fusion gene was not detected (Table 1). Normalized *PML-RARα*/*ABL1* by qRT-PCR was 100% (Figure 2E). The patient was diagnosed with APL, and evaluated as low risk, therefore he was treated by ATRA and ATO without chemotherapy for three courses. Then he treated with ATO alone for five courses due to ATRA intolerance. He achieved molecular remission (MR) 3 months after diagnosis and remained MR status.

**TABLE 1:** Laboratory features of the patient at diagnosis and at secondary leukemia.

	First diagnosis (January 2019)	Secondary leukemia (November 2021)
WBC/Hb/PLT (×10^9^/L/g/L/×10^9^/L)	1.0/95.0/20.0	28.6/143.0/204.0
Morphology	APL	ALL
Immunophenotype	The blasts expressed CD45dim, CD117, CD33, CD38, CD13, CD64 and CD15; no lymphoid antigens were expressed	The blasts expressed CD45dim, CD34, CD10, CD19, CD13, CD22, CD81, CD58, HLA-DR, CD38 and cTdT, partially expressed CD33 and cCD79a, none expressed CD7, CD117, CD9, CD56, CD15, CD20, CD79b, MPO, clgM
Cytogenetics	46,XY,t(15; 17)(q22; q21)	47, XY, +der(22)t(9; 22)(q34; q11)
Molecular biomarkers		
PML-RARα	Positive	Negative
BCR-ABL1	Negative	Positive
IKZF1 gene deletion	Negative	Positive
EP300 mutations	Negative	Positive
JAK3 mutations	Negative	Positive

Note: WBC, white blood cells; Hb, hemoglobin; PLT, platelets; AML, acute myeloid leukemia; ALL, acute lymphoblastic leukemia.

In November 2021, the patient’s laboratory examinations excluded relapse of APL but showed B-ALL (Figure 2A; Table 1). The marrow aspirate showed 51% blasts which expressed CD45dim, CD34, CD10, CD19, CD13, CD22, CD81, CD58, HLA-DR, CD38 and cTdT, partially expressed CD33 and cCD79a, none expressed CD7, CD117, CD9, CD56, CD15, CD20, CD79b, MPO, clgM (Figure 2B). Cytogenetic studies of BM revealed a 47, XY, +der (22) t (9; 22) (q34; q11) karyotype. Fluorescence *in situ* hybridization (FISH) analysis (GP Medical, Beijing, China) showed that 83.5% of examined cells possessed *BCR* (green) and *ABL1* (red) fusion signals (yellow) (Figures 2D, E). Quantitative RT-PCR can also detect *BCR-ABL1* p190 fusion gene (Figure 2E). RNA-sequencing results demonstrated the patient had *IKZF1* gene deletion (*IK6*, exon4-exon7 del), *EP300* (exon31:c.C5957T: p.P1986L) and *JAK3* (exon11:c.G1503T:p.Q501H) mutations. Then the patient was treated by chemotherapy with tyrosine kinase inhibitors (TKI) dasatinib (100 mg/day). The induction chemotherapy regime was VICD (Vincristine, Demethoxydaunomycin, Cyclophosphamide and Dexamethasone). After the induction chemotherapy, the patient achieved a complete hematologic response, while FISH showed that 0.1% of examined cells still expressed the *BCR-ABL1* fusion signals, and molecular biology showed a considerable reduction in *BCR-ABL1* (p190)/*ABL1* from 100% to 0.16% (Figure 2E). The patient’s condition was improved, and he was released from the hospital. After that, the patient received further consolidation chemotherapy of CAM (Cyclophosphamide, Cytarabine and Mercaptopurine) and HD-MTX (high dose methotrexate) with continued usage of dasatinib at 100 mg/day. In April 2022, he underwent allogeneic hematopoietic stem cell transplantation (mother to son, HLA 5/10). During the follow-up, the patient survived and continued to obtain complete molecular biological remission.

In order to determine the specific time of the patient’s secondary leukemia, we retrospectively tested his BM samples before November 2021, and the results showed that the positive expression of the *BCR-ABL1* (p190) gene could be detected as early as August 2021. However, the patient’s blood counts and bone marrow characteristics was normal at that time, and the *PML-RARα* gene was also negative, so the chemotherapy regimen was not changed.

## Discussion

Since the combination of ATRA and ATO with or without chemotherapy, the survival rates of APL patients have been dramatically improved, exceeding 80%–95% (Kantarjian et al., 2021; Ferrara et al., 2022). However, some patients developed therapy-relate myeloid neoplasms (t-MN), such as therapy-related AML or myelodysplastic syndrome (t-AML/MDS), with an incidence of 1%–9.8% (Gaut et al., 2018). And others developed secondary solid tumors (Huang et al., 2001; Au et al., 2007; Eghtedar et al., 2015). Secondary lymphoid neoplasms following treatment for APL were rare, and only four cases have been reported. One case developed precursor T-lymphocytic lymphoma (Szotkowski et al., 2009), two cases developed T-ALL (Liso et al., 1998; Bee et al., 2004), and one case developed early pre-B ALL with *MLL/AF-1p* fusion gene (Tsujioka et al., 2003). To our knowledge, the case we reported here was the first one who developed a secondary ALL with *BCR-ABL1* fusion gene. The *BCR-ABL1* fusion protein is sensitive to TKI, such as dasatinib (Shen et al., 2020), and the patient we reported achieved MR after 3 months of TKI and chemotherapy.

There are many hypotheses regarding the mechanism of secondary malignant neoplasms after successful treatment of APL. One of the hypotheses recognized by the majority of people is that APL induces therapy-related malignancies due to exposure to cytotoxic drugs during treatment (Wang Z. et al., 2019). While it is still unclear which drug may lead to it. In our case, the patient only received ATRO and ATO, without any other chemotherapy. ATRO and ATO maybe the possible reason. The underling possible mechanism is clonal selection. After exposure to APL treatment, the preexisting somatic mutation in hematopoietic stem cell may development to secondary neoplasms. The preexisting somatic mutations mainly tend to DNA damage, such as *TP53* or *PPM1D* mutations (Martin et al., 2020). Moreover, there were also some cases about co-expression of t(15; 17) and other chromosome translocations, such as t(8; 21) (Uz et al., 2013). While the incidence of chromosomal rearrangements in addition to t(15; 17) was rare, and the role of additional translocations was still unclear. The current literature reviews tended to similar prognosis between additional abnormality and t(15; 17) alone. We detected the next-generation sequencing and chromosomal examination of this case before treatment, but no additional rearrangements or mutations were positive. The other possible reason is lineage switch. However, “Lineage switch” is a term used to describe the phenomenon of acute leukemias that meet standard criteria for a specific lineage (either lymphoid or myeloid) at the time of initial diagnosis, but later switch to another lineage upon relapse, including changes in cell morphology, histology, and immunotype (Rossi et al., 2012). While in our case, we detected the fusion gene of *BCR/ABL* at initial diagnosis which was negative, so the possibility of lineage switch was small. Furthermore, the *PML-RARα* fusion gene in our case remained continuous negative, and neither the clinical symptoms nor laboratory data exhibited typical features of APL. We supposed that this secondary ALL might be related to therapy.

To analyze the characteristics of secondary malignancies after APL treatment, we conducted a literature search on PubMed with the keywords “secondary” or “therapy-related”, combined with “after acute promyelocytic leukemia”, “following acute promyelocytic leukemia” to gather related case reports (Eghtedar et al., 2015; Huang et al., 2001; Au et al., 2007; Szotkowski et al., 2009; Liso et al., 1998; Bee et al., 2004; Tsujioka et al., 2003; Wang Z. et al., 2019; Gong et al., 2021; Vicente-Ayuso et al., 2017; Imagawa et al., 2010; Garcia-Manero et al., 2002; Renneville et al., 2018; Athanasiadou et al., 2002; Tang et al., 2016; Dang et al., 2014; Drake et al., 2003; Lee et al., 2005; Bao et al., 2009; Kelemen et al., 2012; Pawarode et al., 2006; Bseiso et al., 1997; Panizo et al., 2003; Snijder et al., 2008; Stavroyianni et al., 2000; Chen et al., 2012; Jubashi et al., 1993; Au et al., 2001; Todisco et al., 1995; Hatzis et al., 1995; Ojeda-Uribe et al., 2012; Meloni et al., 1997; Sawada et al., 1999; Park et al., 2014; Zompi et al., 2000; Liu et al., 2017; Latagliata et al., 2002; Sahoo et al., 2013; Batzios et al., 2009; Lobe et al., 2003; Wang T. et al., 2019; Annunziata et al., 2003; Park et al., 2008; Asou et al., 2010; Felice et al., 1999; Kim et al., 2014; Miyazaki et al., 1994; Takeshita et al., 2004), and combined with our case. A total of 100 cases were included in this literature review. The patients were divided into five groups according to the types of secondary malignancies. Details of age, gender, duration from APL to secondary malignancies, karyotype, and survival time of the patients are shown in Table 2. Among these cases, 46 cases were male, 45 cases were female, and 9 cases were unknown. The age of the patients ranged from 15 to 76 years old (median 48 years old). The median duration from APL to secondary malignancies was 23 (12–168) months. Most of the patients have karyotype changes after secondary disease and often have poor prognosis. Therefore, multiple laboratory testing methods should be combined for early detection of secondary malignancies.

**TABLE 2:** Characteristics of secondary malignancies after APL treatment.

Groups	Gender[N (Female/Male/NA)]	Age[years,M (range)]	Treatment protocol for APL (CT/ATRA+CT/ATRA+ATO±CT/NA)	Number of patients with secondary Cancer subtypes	Duration from APL to SM [months, M (range)]	Karyotypic changes at secondary malignancy (No/Yes/NA)	Median survival time after secondary malignancy (months)	Outcome (Alive/Died/NA)
t-MDS	49(20/21/8)	52(26–73)	4/34/2/9	1/5/2/2/23/16^a^	35.5 (13–180)	5/43/1	16.8 (0.8–184)	8/23/18
t-AML	28(15/12/1)	46(5–81)	2/18/7/1	1/1/6/5/6/9^b^	37 (7–129)	6/22/0	10.8 (1.7–104.4)	7/16/5
t-ALL	4(2/2/0)	25(17–57)	1/2/1/0	2/2^c^	17 (12–33)	1/3/0	8 (7–9)	1/3/0
t-ST	12(7/5/0)	51(11–74)	1/6/5/0	2/1/1/2/1/1/1/1/2^d^	60 (16–125)	7/5/0	22(-)	3/0/9
Others	6(1/5/0)	56(8–61)	3/3/0/0	1/1/1/1/1/1^e^	41 (23–93)	0/6/0	13 (5–35)	2/2/2
Total	99(45/45/9)	48(5–81)	11/63/15/10	NA	36 (7–180)	19/79/1	12 (0.8–184)	21/44/34

Note: APL, acute promyelocytic leukemia; MDS, myelodysplastic syndrom; AML, acute myeloid leukemia; ALL, acute lymphoblastic leukemia; ST, solid tumor; CT, chemotherapy; ATRA, all-trans retinoic acid; ATO, arsenic trioxide; M, median; NA, not available.

a, 5q-/RA/RARS/RCMD/RAEB/NA.

b, M0/M1/M2/M4/M5/NA.

c, B-ALL/T-ALL.

d, Breast/Prostate/Vulvar/Colon/Soft tissue sarcoma/Melanoma/Pancreatic/Renal/Nasopharynegeal.

e, T-lymphoblastic lymphoma/Chronic myelomonocytic leukemia/Atypical chronic myelocytic leukemia/Biphenotypic leukemia/Acute Leukemia without subtype/myeloid neoplasm.

In conclusion, we reported a rare case of secondary *BCR-ABL1* positive ALL after successful APL treatment. The patient responded well to TKI and chemotherapy and achieved a MR. Although APL usually has a good prognosis, the therapeutic effects of secondary malignant tumors are different. At present, there are no effective measures to prevent the occurrence of secondary tumors. For this subset of patients, the monitoring frequency of molecular biomarkers (not only *PML-RARα*) should be increased after receiving CR, in order to achieve the purpose of early detection and early treatment.

## Data Availability

The original contributions presented in the study are included in the article/Supplementary Material, further inquiries can be directed to the corresponding author.
